# Three-dimensional histological specimen preparation for accurate imaging and spatial reconstruction of the middle and inner ear

**DOI:** 10.1007/s11548-013-0825-7

**Published:** 2013-04-30

**Authors:** Thomas S. Rau, Waldemar Würfel, Thomas Lenarz, Omid Majdani

**Affiliations:** Department of Otolaryngology, Institute for AudioNeurotechnology (VIANNA), Hannover Medical School, Carl-Neuberg-Str. 1, 30625 Hannover, Germany

**Keywords:** Microgrinding, 3D Imaging, Reconstruction, Histological sectioning, Modelling, Cochlea, Middle ear, Temporal bone

## Abstract

**Purpose:**

This paper presents a highly accurate cross-sectional preparation technique. The research aim was to develop an adequate imaging modality for both soft and bony tissue structures featuring high contrast and high resolution. Therefore, the advancement of an already existing microgrinding procedure was pursued. The central objectives were to preserve spatial relations and to ensure the accurate three-dimensional reconstruction of histological sections.

**Methods:**

Twelve human temporal bone specimens including middle and inner ear structures were utilized. They were embedded in epoxy resin, then dissected by serial grinding and finally digitalized. The actual abrasion of each grinding slice was measured using a tactile length gauge with an accuracy of one micrometre. The cross-sectional images were aligned with the aid of artificial markers and by applying a feature-based, custom-made auto-registration algorithm. To determine the accuracy of the overall reconstruction procedure, a well-known reference object was used for comparison. To ensure the compatibility of the histological data with conventional clinical image data, the image stacks were finally converted into the DICOM standard.

**Results:**

The image fusion of data from temporal bone specimens’ and from non-destructive flat-panel-based volume computed tomography confirmed the spatial accuracy achieved by the procedure, as did the evaluation using the reference object.

**Conclusion:**

This systematic and easy-to-follow preparation technique enables the three-dimensional (3D) histological reconstruction of complex soft and bony tissue structures. It facilitates the creation of detailed and spatially correct 3D anatomical models. Such models are of great benefit for image-based segmentation and planning in the field of computer-assisted surgery as well as in finite element analysis. In the context of human inner ear surgery, three-dimensional histology will improve the experimental evaluation and determination of intra-cochlear trauma after the insertion of an electrode array of a cochlear implant system.

## Introduction

**Fig. 1:**
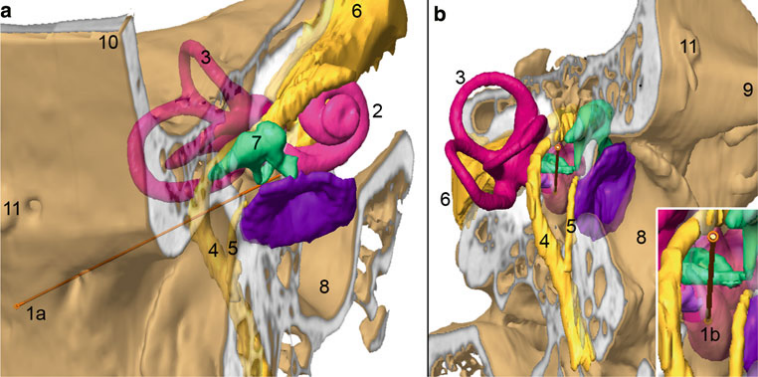
Preoperative planning founded on flat-panel-based volume computed tomography (fpVCT) imaging. **a** Slightly superior frontal view of the lateral skull base. **b** Lateral view, slightly posterior and inferior, showing a planned trajectory (*1a*–*1b*) for minimally invasive access to the inner ear (*2*, cochlea). **a**, **b** CT-based imaging allows the identification of the functional structures of the lateral skull base: cochlea (*2*), vestibular system (*3*), facial nerve (*4*), chorda tympani (*5*), vestibulocochlear nerve (*6*) and ossicular chain (*7*). Additionally, the external ear canal (*8*) with tympanic membrane, the zygomatic process of the temporal bone (*9*), squama temporalis (*3*) and artefacts of a bone-anchored screw (*11*) serving as fiducial markers for the registration procedure are visible. Apart from the ossicles, all structures are segmented indirectly using the high contrast of their bony walls. Using these patient-specific anatomical data, a trajectory serving as a minimally invasive access to the inner ear can be planned, starting at the surface of the temporal bone (*1a*) and entering the cochlea near the round window membrane (*1b*). However, intra-cochlear structures such as the basilar membrane which are essential to planning the insertion of a cochlear implant electrode are not visible

Image-guided surgery (IGS) systems^1^ or medical robots are gaining in clinical importance due to the increasing requirements for accuracy and safety of surgical interventions. These approaches in computer-assisted surgery (CAS) are mostly based on a three-dimensional (3D) image representation of the patient-specific anatomy as a necessity for preoperative planning and intraoperative implementation (Fig. 1). Hence, there are also growing demands in terms of adequate medical imaging. Especially in domains where high-resolution soft tissue imaging is necessary, imaging is a limiting factor for the implementation of a highly accurate computer-assisted surgical approach.

The implantation of a cochlear implant (CI) system for the treatment of patients who suffer from severe hearing loss or deafness is just such a surgical intervention which imposes high demands on intraoperative accuracy. There are various reasons why CI surgery seems to be predestined for computer-assisted improvements regardless of whether the research is focussed on one or more of the steps involved in CI surgery, such as:

(i) milling the implant bed [24, 25, 72];
(ii) performing the mastoidectomy [35, 36, 73, 98, 99, 116];
(iii) performing the cochleostomy through a minimally invasive access [5–7, 20, 23, 51–53, 58, 64–66, 114]; or
(iv) automated insertion of the electrode array [42, 67, 79, 90, 91].

First, CI treatment is characterized by a high degree of social relevance owing to the large number of affected people and the central importance of hearing for human communication and thus social interaction. Improvements in hearing substantially affect the quality of life [19] and directly depend on the operative outcome. In addition, the indication for cochlear implants is changing; whereas it used to be total deafness, increasingly patients with residual hearing are being considered on the strength of motivating results with combined electrical and acoustic stimulation [10, 30, 60, 74, 104, 109, 110]. Thus, new challenges have to be tackled. In particular, the requirements for accuracy grow in the context of an atraumatic opening of the cochlea and electrode insertion. Furthermore, the lateral skull base shows a high complexity of functionally important anatomical structures which impose high demands on surgical precision and safety. Therefore, mechatronic devices and intraoperative visualization support the prevention of iatrogenic damage of endangered structures and also help to securely identify anatomical landmarks.

**Fig. 2:**
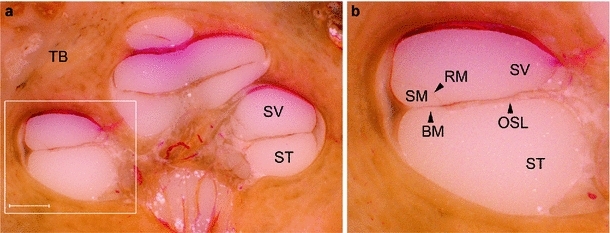
Intra-cochlear anatomical structures. **a** Histological microgrinding preparation of the human inner ear after embedding in epoxy resin (dyed with titanium dioxide, $\text{ TiO }_{2}$) and acid violet staining of the soft tissues. **b** Close-up of the basal turn. An electrode array of a cochlear implant system needs to be inserted into the scala tympani (*ST*) without harming the basilar membrane (*BM*) or other internal structures. *TB* surrounding temporal bone, *NC* nervus cochlearis, *SV* scala vestibule, *SM* scala media, *RM* Reissner’s membrane, *OSL* Osseous spiral lamina. (TB1R, *scale bar* is 1 mm)

However, while procedures (i) and (ii) can be achieved by conventional radiological imaging, (iii) and especially (iv) approach the limits of currently available modalities. For these, adequate imaging of soft tissue structures is necessary for preoperative planning. During both the drilling of the cochleostomy and the insertion of the electrode array, no essential membranous structures within the cochlea should be destroyed. In particular, the preservation of the basilar membrane is crucial to preserve the residual hearing (see Fig. 2). The outer and inner hair cells are located on this delicate structure. They are the central functional elements of natural hearing, transducing acoustic information into auditory nerve response. The basilar membrane is situated approximately in the middle of the inner ear (cochlea), dividing the fluid-filled lumen and separating the scala tympani. It is of major interest in cochlear implant surgery as it is the preferred location for the electrode array. Thus, using computer-aided surgery to improve the precision and safety of the intervention, the shape and location of the scala tympani—which is partly bound by membranes—must be accurately known during imaging.

Additionally, development of cochlear implant electrodes on the basis of simulation and computer-aided engineering (CAE) is currently still rather uncommon. There are few simulation-based approaches to evaluating the available CI electrodes in terms of intra-cochlear movement and positioning [18, 46, 47]. Therefore, besides the minimally invasive approach to the cochlea, another aim of our research is to establish computer-aided design (CAD) in combination with simulation in the field of cochlear implant electrode development. Using finite element analysis (FEA), forces between implant and intra-cochlear tissues, such as the bony wall or the basilar membrane, will become calculable. Therefore, more complex and detailed models of the cochlear anatomy become necessary, which are based on adequate imaging that includes all essential soft tissue structures.

The challenge is to achieve high-resolution visualization of both high-contrast structures such as bone and soft tissue with nearly equal attenuation coefficients in close proximity. Since clinically available multislice computed tomography (MSCT), digital volume computed tomography (DVT) and magnetic resonance imaging (MRI) do not offer the required high spatial resolution and/or soft tissue differentiation, several imaging methods have been investigated. These experimental approaches include flat-panel-based volume computed tomography (fpVCT) and micro-computed tomography (micro-CT or $\upmu \text{ CT }$) as improvements on conventional CT imaging, magnetic resonance microscopy (MRM) as an experimental approach based on MRI, different (laser) light-sheet-based imaging methods such as orthogonal plane fluorescence optical sectioning (OPFOS) and optical coherence tomography (OCT), as well as different techniques of serial-section imaging. In this context, the literature often presents the basilar membrane as a benchmark for an adequate imaging outcome because it combines functional importance with the challenge of a thin soft tissue structure.

An important step in this context was the introduction of micro-computed tomography which has proven to be a suitable tool particularly with regard to high-density and high-contrast structures, such as the ossicular chain [21, 54, 55, 77, 84, 95, 106]. For the visualization of small structures, however, the thickness of the surrounding bone has to be minimized by specimen preparation because it absorbs a lot of radiation necessary for high-resolution imaging of interior structures [21]. Another option would be partial or complete decalcification of the bony structures [75, 80] or the use of less calcified foetal specimens [93]. However, achieving adequate imaging of soft tissue structures within the middle and inner ear is still a challenge and will require additional effort [68, 92]. The simplest approach is the optimization of scanning parameters with a special focus on soft tissue structures. Reduction tube voltage, for example, causes improved greyscale differences between soft tissue structures of the middle ear and background air [92, 95]. Moreover, long integration time and very low resolution would appear to enable the slight visualization of the basilar membrane as recently shown by Braun et al. [9]. Another possibility is the use of contrast agents. The injection of osmium tetroxide ($\text{ OsO }_{4})$, for instance, was used to improve the visibility of the membranous labyrinth of the inner ear [76, 105]. In addition to the toxic osmium tetroxide, the use of low-toxicity contrast agents is reported for the imaging of vertebrate embryos [69], various animal tissues [68] and recently also for the human middle ear [2]. Alternatively, Postnov et al. [75] replaced the perilymph (the fluid which normally fills the scala tympani and vestibuli) with air to increase the contrast between the background and the basilar membrane for improved visualization.

Another effective way to improve the quality of X-ray imaging is the use of coherent hard X-rays from a synchrotron radiation source [31, 33, 56, 80] instead of portable scanners with their own X-ray source. This allows imaging of thin membranes even down to the subcellular range. The obvious drawback is the highly restricted availability of synchrotron facilities [68].

In addition, MRI was investigated as it offers obvious advantages in tissue differentiation in comparison with micro-CT imaging. Using high-field strength MR systems (additional local coils) and long acquisition time, resolution could be improved down to the micrometre range. Because the micrometre range is involved, these techniques became known as MRM. Beginning with Henson et al. [34] in 1994, 3D magnetic resonance microscopy was reported as an experimental method for imaging intra-cochlear soft tissue structures [34, 49, 55, 85]. When it was applied, the visualization of the basilar membrane with a thickness of about $100\,\upmu \text{ m }$ and even of Reissner’s membrane (approx. $10\,\upmu \text{ m }$ thick) became possible [49].

Another approach to visualizing anatomical structures non-destructively in high resolution is based on virtual optical sections. To generate these images, a thin laser light sheet is used and the omnidirectional fluorescence of the chemically treated specimen is recorded orthogonally [17]. This requires transparency of the object, either by using the natural transparency (as with some larvae or embryos [40, 41]) or by performing a chemical process (termed clearing) to render the sample translucent. Through rotation or translation of the specimen, series of 2D images from different directions or depths can be obtained and used for 3D reconstruction [37–39, 88, 107, 108]. Voie et al. [107, 108] were the first to publish this new microscopic imaging technique. They called their method orthogonal plane fluorescence optical sectioning microscopy (OPFOS) and used it for visualization of the cochlea. Subsequently, improved middle and inner ear models were provided by Santi et al. [88] and Buytaert et al. [16] using OPFOS. Up to now, a couple of related or improved techniques have been developed and published under various names, including: selective plane illumination microscopy (SPIM, [40]), multidirectional SPIM (mSPIM, [41]), high-resolution (hr-)OPFOS [14–16], digital scanned laser light-sheet microscope (DSLM, [45]), ultramicroscopy [22] and thin-sheet laser imaging microscope (TSLIM, [44, 87]). All these different implementations are covered by the acronym LSFM, which stands for light-sheet-based fluorescence microscopy. Excellent reviews are provided by [15, 17, 41, 86].

In the context of FEA of the middle ear including soft tissue structures, histological section preparation is used [27–29] to overcome the above-mentioned drawbacks of conventional imaging. Sun et al. [101] published a very detailed and systematic description. This method was also used in several subsequent studies [27–29, 102]. For a detailed reconstruction of the human middle ear, they decalcified a freshly extracted human temporal bone and fixed it in celloidin. Perpendicular fiducial holes were drilled for image alignment, and finally, the probe was serially sectioned. Sequential slices with a distance of 200 $\upmu \text{ m }$ were used for 3D reconstruction. Wang et al. [112] applied the same preparation procedure but aligned consecutive images by means of an intensity-based registration algorithm instead of using reference markers.

Another detailed procedure description was published by Sørensen et al. [96] and performed for the visible ear project. Again, the tissue block (covering the temporal bone) was provided with vertical drill holes serving as position markers and afterwards sectioned in increments of $50\,\upmu \text{ m }$. Image registration was performed by a least-square fitting algorithm on the registration markers. A more detailed overview on selected studies is provided in Tables 4 and 5.

These previously mentioned studies suggest that it is possible to completely reconstruct the complex anatomical structure of the temporal bone, including thin soft structures. However, $\upmu \text{ CT }$ imaging is still limited in terms of soft tissue visualization, MRM does not provide the requisite resolution, and optical imaging methods such as OPFOS require a complex chemical procedure which influences the dimensional distortion of the tissue. Finally, commercial micro-CT scanners are expensive, and MRM and LSFM require custom-made experimental set-ups whose realization depends on wide experience obtained over several years.

An improved serial-sectioning method, based on our long-standing experience of the microgrinding procedure of the rigid fixation in epoxy resin, seems to have advantages regarding soft tissue visualization. Nevertheless, it still is a complex procedure dealing with several challenges, of which the minimization of dimensional distortion is the most demanding, as mentioned by Ferguson et al. [26]. This dimensional stability constitutes the precondition for geometrically accurate preservation of the anatomical structures, as the necessary preparation processes are destructive. The most remarkable fact about the above-mentioned studies using histological sectioning is that they do not include an investigation of the quality and accuracy of the performed 3D reconstruction. Therefore, it remains questionable whether these methods are really appropriate for accurate and realistic imaging, especially in the context of surgical interventions.

The motivation behind our study is to overcome these deficiencies. Since none of the clinically available imaging techniques offers either the desired spatial resolution or detailed soft tissue differentiation, we pursue a model-based imaging approach [89]. This involves combining standard medical imaging (e.g. MSCT) with digital anatomical models provided by a highly accurate imaging method which visualizes necessary soft tissue structures. By merging these data (which means warping the anatomical model to match the patient-specific images), clinical imaging can be completed by means of additional relevant information from a database containing averaged anatomical data.

For this reason, the aim of the present study is to provide a highly accurate preparation and 3D reconstruction procedure which is necessary for the further establishment of an anatomical database of this kind. As will become apparent, the method allows the dimensionally stable sectioning of relevant anatomical structures of the middle and inner ear (including the ossicular chain, corresponding muscles, tendons and ligaments) as well as of the membranous inner ear (including the stria vascularis, basilar membrane and scala tympani). Additionally, the accuracy of the 3D reconstruction method was investigated for the first time using a geometrically well-known reference object. Furthermore, reconstructed image stacks of embedded temporal bone specimens were merged with the corresponding imaging data from high-resolution computed tomography (fpVCT) to show the accuracy of the histological microgrinding procedure.

## Materials and methods

### Specimen preparation

**Fig. 3:**
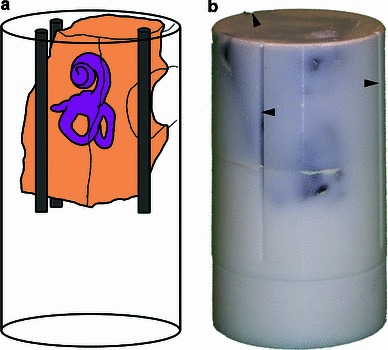
Embedding of the temporal bone specimen. **a** Schematic drawing of the embedded temporal bone specimen showing its location within the cylindrical sample of epoxy resin as well as three artificial registration markers. **b** The photograph of the hardened sample shows the three longitudinal grooves (*black markers*) milled on the surface for accuracy measurement

The study was performed using 12 freshly dissected human temporal bone specimens^2^ which were cleaned from surrounding tissue by means of a bone saw (Labotom-2, Struers A/S, Ballerup, Denmark). In this way, a cuboidal sample was produced containing the middle and inner ear (Fig. 3a), whose dimensions respect the spatial limitations of the silicone mould used in a subsequent step. During sample preparation, one of the semi-circular canals was cut to allow the infiltration of the epoxy resin into the (otherwise unharmed) labyrinth. After trimming, the specimen was immediately placed into phosphate-buffered (0.1 mol/L, pH 7.4) 4 % glutaraldehyde in which it was stored for 4 h. The subsequent rinsing in a phosphate buffer lasted another 2 h. After that, a dehydration process was performed using a five-step ethanol series with increasing concentrations (resting time and respective concentration: 3 h in 50 %, at least 14 h in 70 %, another 3 h each in 90 and 96 %). Finally, the specimen was placed in an equal mixture of alcohol and acetone for 3 h. The embedding preparation was completed by desiccating the specimen in a drying cabinet (60 $^{\circ }\text{ C }$) for 16 h.

Afterwards, the fixed and dehydrated sample was put in a cylindrical silicone mould (inner diameter approx. 30 mm, height: 40 mm), which was filled with freshly prepared epoxy resin consisting of 25 parts of resin, three parts of hardening agent and 0.5 parts of thinner (Epofix and TekMek, Struers A/S, Ballerup, Denmark). Titanium dioxide ($\text{ TiO }_{2})$ was added as colourant, making the epoxy resin non-transparent to ensure the invisibility of underlying structures and hence facilitate improved identification of anatomical structures at each grinding surface. The epoxy-filled mould was then immediately placed into a vacuum cabinet (Vacuubrand RE8, Vacuubrand, Wertheim, Germany) for five minutes to eliminate gas bubbles from the specimen’s inner cavities, thus ensuring homogeneous infiltration of the resin. After releasing the vacuum, the epoxy resin was hardened at room temperature for at least 8 h.

After hardening, cylindrical turning was performed to allow optimal fixation of the epoxy block in a custom-made specimen holder. Additionally, three longitudinal grooves were milled onto the block (Fig. 3b) to serve as reference objects in CT imaging.

### CT imaging

All embedded specimens were scanned in an experimental fpVCT (GE Global Research Niskayuna, NY, USA) at the Department of Diagnostic Radiology at Goettingen University Hospital. The CT scanner consists of an X-ray tube and two amorphous-silicon flat-panel detectors, both consisting of $1.024 \times 1.024$ detector elements on an area of 20.48 cm $\times$ 20.48 cm. Therefore, the physical resolution amounts to 200 $\upmu \text{ m } \times 200\,\upmu \text{ m }$ in the x-y plane. A tube voltage of 140 kV was applied together with a current of 20 mA. These scanning parameters correspond to the values used throughout our former studies involving fpVCT [8, 64, 65] and were successfully established for our research on image-guided and robot-assisted temporal bone surgery. During a $360^{\circ }$ rotation of the detector element performing the one-detector mode, 1,000 projections were acquired. The cone-beam covers a scan field of 13.5 cm in diameter and 4.21 cm in the z-direction. The latter can be increased by performing a step-and-shoot scanning shot and stacking the acquired volumes. During reconstruction, using a $512 \times 512$ pixel matrix and interpolation, the field of view was cropped to the size of the specimens. Finally, the data set was exported to the Dicom3 format.

### Preparing serial microgrinding

**Fig. 4:**
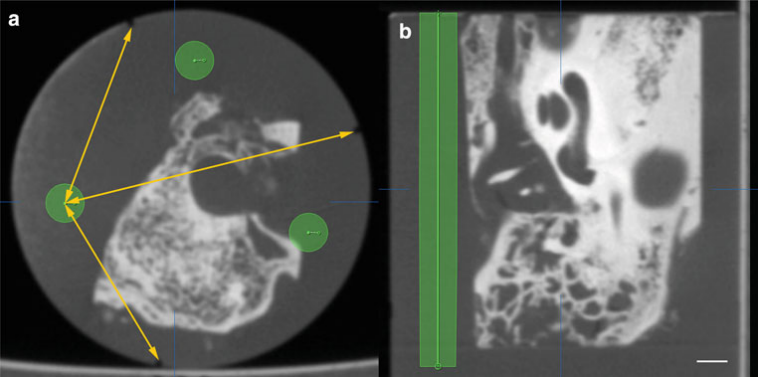
Planning of the registration markers. Trajectory planning allows the definition of proper locations for the three artificial registration markers avoiding damage of essential structures of the specimen (**a**: axial view, **b**: sagittal view). Triangulation (symbolized by the *yellow arrows*), using the milled grooves on the surface as reference points, allows the transfer of the planned locations for the registration markers to the base area of the sample. The *scale bar* is 2 mm

After scanning, three fiducial markers were added to the sample to ensure accurate image registration using a feature-based approach. The previously acquired fpVCT data sets were consulted to determine the proper locations for the drillings in order to prevent damage to essential structures of the embedded temporal bone. Therefore, each corresponding data set was loaded into iPlan ENT 2.6 using PatXfer 5.2 (both BrainLAB AG, Feldkirchen, Germany). The provided “trajectory planning” function was used to define the locations of three longitudinal drilling holes with an outer diameter of 3.2 mm (Fig. 4). This measure corresponds to cylindrical plastic pins (LEGO$^\circledR$ Group, Billund, Denmark) chosen as artificial registration markers. With the aid of what is known as the “probe view”, showing a layer perpendicular to the planned drilling path, it was possible to estimate the distance to essential anatomical structures with a view preserving them. Surface coordinates of the longitudinal drilling path were transferred from the planning tool to the real sample via triangulation of the distances between their centres and the milled grooves as reference points on the circumference of the sample (Fig. 4a).

Thus prepared, the sample was secured in the custom-made specimen holder (see below). A computer numerically controlled (CNC) machine was applied to ensure perpendicular drilling of the holes. This procedure also provides the accurate distances between each hole, which were subsequently used for scaling the images. Finally, the drill holes were filled with black plastic pins to provide the necessary high contrast for an automated image alignment algorithm [78]. The application of this algorithm allows the simplified correction of different positions and orientations of the sample in the microscope’s field of view, as well as corrections of slight differences in size caused by manual handling and focusing.

### Highly accurate microgrinding procedure

**Fig. 5:**
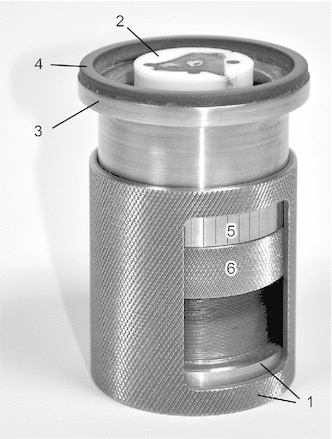
Custom-made specimen holder for controlled manual grinding. The special grinding tool consists of two main parts. The inner part (*1*) secures the embedded specimen (*2*). The outer part (*3*) is equipped with an abrasion-resistant hard ceramic ring (*4*) which defines the grinding surface. An added scale (*5*) allows easy adjustment of a specific amount of abrasion and a look ring (*6*) ensures preservation of the setting during the grinding process

For accurate 3D reconstruction, the distance between each serial image of the stack has to be known. As this aspect was considered by us to be a main source of error, a special grinding tool was developed [78] which allows the setting of a specific amount of abrasion. The specimen holder consists of two parts (Fig. 5), securing the embedded specimen at its distal end within the inner part. The second part of the specimen holder serves as a bush and is equipped with an abrasion-resistant hard ceramic ring. Via a fine thread, the bush can be threaded backwards so that a defined part of the sample becomes uncovered and available for abrasion. This mechanism allows the setting of a well-defined abrasion within the range of a few micrometres. For further improvement of accuracy, each actual abrasion was measured and documented using a tactile measuring device (Heidenhain Specto ST 3048, Heidenhain GmbH, Traunreut, Germany), with 2 $\upmu \text{ m }$ resolution. The decision to incorporate this additional measurement step is based on several reasons (see the Discussion section for further details). In summary, it allows control of each layer’s correct abrasion. Detected insufficient abrasion was corrected by a further grinding step. Additionally, the measured abrasion was incorporated when reconstructing the 3D image stack. As has been shown in our previous study [78], this method greatly improves the overall accuracy of the 3D reconstruction in contrast to applying the defined abrasion mechanically determined by the specimen holder (Fig. 5).

For abrasion, $100\,\upmu \text{ m }$ steps were chosen between cross-sectional images. This interval was chosen as a compromise between the resulting voxel size and the time factor, since the entire procedure was carried out manually without additional automation. Grinding was performed using a commercial grinding machine (LaboPol-5, Struers A/S, Ballerup, Denmark). The specimen holder was manually pressed on the water-cooled grinding disc. The commercially available specimen mover LaboForce-1 (Struers A/S) was replaced by attaching a custom-made guide ring which is suitable for the outer diameter of the specimen holder. This additional support during manual holding and pushing improves coplanar abrasion. The holder was pressed until abrasion was no longer observable. To achieve a polished surface, fine sandpaper with a constant grit size of P800 was used (Buehler GmbH, Düsseldorf, Germany).

Afterwards, the specimen’s dry surface was stained with acid fuchsin (acid violet 19, CAS No: 3244-88-0) for approximately 45 s, resulting in violet staining of the superficial soft tissue structures. The procedure was completed by carefully rinsing and air drying one last time. Subsequently, the surface was wetted with a few drops of distilled water and covered bubble-free with a cover glass to improve image quality. Imaging was performed with the Leica APO Z6 macroscope (Leica Microsystems GmbH, Wetzlar, Germany) with full apochromatic optic (objective $0.5\!\times$ Apo, Z6/Z16, $\text{ f } = 187\,\text{ mm }$). The optical resolution of the macroscope is approximately 30 line pairs per millimetre (lp/ mm), which means that the smallest visible structure is in the range of 17 $\upmu \text{ m }$. A five megapixel CCD camera (DFC 420, Leica Microsystems GmbH, resolution: $2.592 \times 1.944$ pixels) was attached to the optical system. It is probable that digitalization slightly degraded the total resolution of the system. The images were stored as eight-bit coloured tiff data files. To brighten up the images, a cold light source (Leica KL 1500 LCD) in combination with a ring light has proven particularly suitable.

The iterative procedure—including adjustment of 100 $\upmu \text{ m }$ abrasion at the specimen holder for the next layer, microgrinding, verifying abrasion, staining and digitalization by means of the macroscope—was repeated until all anatomical structures of interest had been recorded.

### 3D reconstruction and image processing

For automated image registration, an algorithm was developed that detects the added artificial registration markers and, based on these, aligns all images of the stack according to the first (base) image. The algorithm, implemented in Matlab (The MathWorks, Inc., Natick, MA, USA, including the Image Processing Toolbox), has been described in more detail previously [78]. In general, the main principle involves using the colour range of the manually marked registration markers of the base image positions as parameter for a colour filter in the HSV colour space. This procedure is performed on each subsequent image, followed by median filtering, image closing and performing the Hough transform [70] to find circular structures. By means of automated detection, the approximate position of each marker becomes available. This information is used to crop detail images which include the registration markers. Additional processing steps are performed on each of these detail images to further improve the determination of the registration markers’ centre. These steps include edge detection and a second Hough transform [11] followed by the application of a circle fitting algorithm [13] on the remaining pixels. The accurately determined positions of the registration markers are subsequently used for linear conformal image registration.

Finally, the resulting image stack is cropped and limited to the relevant structures. For intermediate quality control and determination of reconstruction accuracy, the detail includes the milled grooves. Afterwards, the image stack is cropped for a second time (see Table 1), now only showing the relevant structures of the middle and inner ear. In so doing, the amount of data is reduced to improve the performance of future data processing such as image fusion and object segmentation.

**Table 1:** Overview of the specimens processed within the study showing the number of slices, size of the resulting image stacks before (image size cut) and after final trimming to the middle and inner ear structures (image size cut min), the achieved resolution (pixel spacing) as well as the size of each Dicom data set

Specimen (side)	Number of slices	Image size cut [$\text{ Px } \times \text{ Px }$]	Image size cut min [$\text{ Px } \times \text{ Px }$]	Pixel spacing [$\upmu \text{ m }/\text{ Px }$]	Size Dicom set [MB]
TB-1L	169	1,276 $\times$ 1,795	1,030 $\times$ 1,102	16.40	366
TB-1R	169	1,489 $\times$ 1,723	1,028 $\times$ 961	15.96	318
TB-3R	185	1,804 $\times$ 1,498	994 $\times$ 880	15.80	308
TB-4L	132	1,624 $\times$ 1,795	890 $\times$ 1,124	16.30	252
TB-4R	175	1,786 $\times$ 1,543	994 $\times$ 1,030	16.30	341
TB-5L	182	1,375 $\times$ 1,633	1,054 $\times$ 913	16.40	334
TB-5R	165	1,647 $\times$ 1,659	1,072 $\times$ 808	16.30	272
TB-6L	171	1,607 $\times$ 1,561	932 $\times$ 972	16.32	295
TB-6R	179	1,702 $\times$ 1,429	1,090 $\times$ 868	16.40	323
Mean	170			16.24	312
Max	185			16.40	366

### Generation of the Dicom file set

**Fig. 6:**
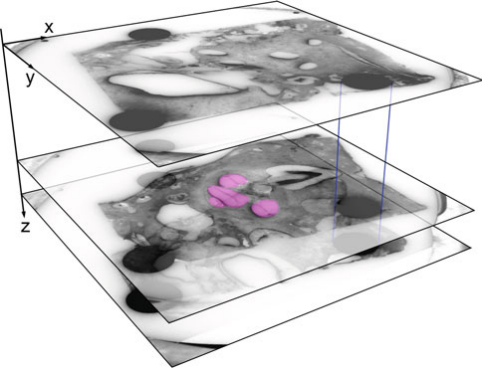
Three-dimensional reconstruction of the cross-sectional images as part of the conversion into the Dicom data set. The artificial registration markers enable accurate image alignment (as indicated by *blue lines*). The measured abrasion distance provides the necessary information to locate each slice in the spatial image stack for an accurate 3D reconstruction of the specimen. Abrasion distances were added cummulatively. For ease of identification, the cochlea is highlighted in *magenta* in this schematic drawing

The aim of the presented image processing procedure is to provide data which can be utilized similarly to regular CT or MRI data, and especially to enable easy image fusion of these different modalities. Therefore, the image stack was converted into the clinically established and wide-spread Dicom standard (Digital Imaging and Communications in Medicine). A Dicom file consists of the actual image data as well as a header containing additional information. The Matlab image processing toolbox provides several functions to generate Dicom files with valid header entries, called attributes, including the unique identifiers (UID) which are mandatory for each Dicom data set. There are also image-specific entries describing the location of the particular image within the stack. The necessary values were calculated as the cumulative sum of the measured abrasion distances, starting with 0 for the first layer (Fig. 6). The accurate scaling of the images was realized by dividing the known distance between two registration markers in millimetres (available from the CNC drilling) and the corresponding distance in the base image in pixels. The average of the three marker distances was set as the “pixel spacing” attribute of each header (Table 1). Additionally, the value for slice thickness was set to the lowest possible level of 0.001 mm to adhere to the two-dimensional character of each grinding image.

### Measurement of accuracy

Before applying the presented method to human temporal bone specimens, its accuracy was evaluated by comparing the reconstructed geometry with the original geometry of a well-known reference object. This was done to determine the preservation of geometric dimensions such as lengths, parallelism, rectangularity and evenness. Due to its low production tolerances, an assembly of LEGO bricks was chosen as a reference object. The measures of this reference object, obtained by a tactile coordinate measuring machine (Zeiss ZMC 550), serve as reference values for comparison.

The reference object was embedded and processed as described above, except for the steps in sample preparation such as trimming, dehydration and drying. Additionally, the epoxy resin was dyed dark blue with “black pigment” (Buehler GmbH, Düsseldorf, Germany), and white registration markers were used instead of the black ones to ensure the same high contrast for the automated marker detection algorithm. Furthermore, straining of the grinding surface was not necessary. Apart from that, the same custom-made algorithms were used for marker detection, image alignment and transfer of the data into the Dicom standard as described above. Finally, the Dicom stack was imported into iPlan 2.6 ENT (BrainLAB AG), and the reference object was segmented using a threshold segmentation algorithm. The resulting volume object was exported by using iPlan’s VVLink interface and converted into the STL format (standard tessellation language) which is a popular format for data exchange between segmentation and FEA software. The dimensions of this “virtual” representation of the reference object—such as length of edges, parallelism and rectangularity of edges and planes—were measured using a custom-made measurement software (courtesy of A. Hussong, Institute of Mechatronic Systems, Leibniz University of Hannover) based on the Visualization Toolkit (Kitware, Clifton Park, NY, USA). This software allows the coordinates of manually selected surface points to be determined. Twenty surface points were selected for each plane, and a best-fit plane was calculated using a least-square algorithm. The edges were calculated via intersection of two corresponding planes, and the corners as the intersection point of three adjacent planes. To determine parallelism and rectangularity of planes and edges, their regular vectors or direction vectors were used. These results were compared with the measurement results of the coordinate measuring machine (CMM) to determine accuracy of the microgrinding and 3D reconstruction procedure in general. (For a more detailed description of this, see our previous publication [78]).

The reconstruction accuracy of the temporal bone specimens was evaluated using the added grooves. They are visible in the fpVCT data as well as in the serial cross-sectional imaging. Using them, enabled the deviation of both data sets to be measured after image fusion. Therefore, the generated data set was imported into iPlan ENT 2.6 using PatXfer 5.2 and added to the already imported fpVCT data of the corresponding specimen. This allows the image fusion of both data sets using the same-named function provided by iPlan. The software applies an implemented intensity-based image registration algorithm to overlap both image stacks. Afterwards, the measurement function of iPlan was used to determine the deviation of the corresponding grooves. Both data sets are visualized using different colours whose intensity can be changed in the overlapping view. Changing the intensity either of the fpVCT data or of the microgrinding images, the grooves could be selected for measuring the distance between them. As measurement accuracy is limited to 0.1 mm-steps, deviations less than 0.05 mm could not be determined.

## Results

The proposed method was successfully performed on the reference object as well as on the embedded temporal bones. First, more general results concerning the accuracy of the introduced reconstruction procedure are presented; afterwards the findings from the temporal bone specimens are reported in detail.

### Accuracy of the 3D reconstruction

Using the geometrically well-known reference object, the accuracy of the serial cross-sectional preparation method was determined as follows: parallelism and rectangularity of an object can be preserved with an average deviation of less than $0.4^{\circ } \pm 0.3^{\circ }$ according to the $0^{\circ }$ angle (parallel edges or coplanar planes) and the $90^{\circ }$ angle (orthogonal edges or planes), respectively [78].

The maintaining of lengths was regarded on a differentiated basis. The reconstruction of edges of an object in the z-direction directly depends on the achieved abrasion, whereas distances within the grinding layer remain unaffected by this. In the z-direction, which was considered to be more crucial, lengths were preserved with an average error of $0.5\,\% \pm 0.4\,\%$. For lengths within the grinding layer, the determined error was, $2.0\,\% \pm 0.4\,\%$. Table 2 summarizes the results. A possible source of error was, presumably, the utilized optical system—a stereo microscope with a non-perpendicular optical path [78]. Hence, the APO Z6 was used for microgrinding preparation of the temporal bone specimen. It is a high-quality optical system especially designed for accurate digital photo documentation and was subsequently purchased because of the initial results using the reference object.

**Table 2:** Accuracy of spatial reconstruction of the serial cross-sectional preparation method

Category	$\text{ Mean } \pm \text{ SD }$	*n*
Length in grinding plane	$2.0\,\% \pm 0.4\,\%$	7
Length in grinding direction	$0.5\,\% \pm 0.4\,\%$	3
Parallelism of planes	$0.4^{\circ } \pm 0.1^{\circ }$	4
Rectangularity of planes	$0.2^{\circ } \pm 0.1^{\circ }$	8
Rectangularity of edges	$0.4^{\circ } \pm 0.3^{\circ }$	30

### Sample preparation

After verifying the accuracy of the introduced serial cross-sectional preparation technique, this procedure was performed on 12 temporal bone specimens. The use of three of them had to be discontinued because CT imaging revealed insufficient embedding of intra-labyrinth structures visible as bubbles in the epoxy resin. Each of the remaining samples underwent the entire microgrinding procedure as described above.

**Fig. 7:**
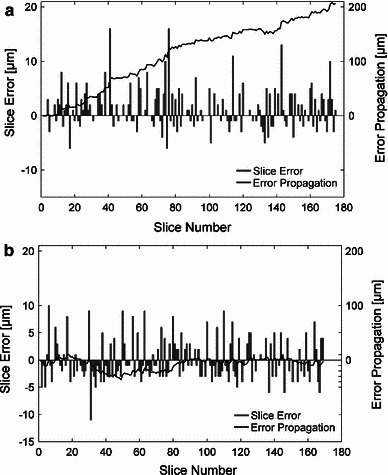
Error propagation of the microgrinding procedure. The difference between the theoretical location of each grinding surface (if equidistant abrasion of 100 $\upmu \text{ m }$ is assumed) and the actually measured abrasion was investigated. The *bar* plot shows the deviation from 100 $\upmu \text{ m }$ abrasion for each grinding surface (slice error). The total error (*line plot*) is a theoretical value determined by the idealized location of the slice as a multiple of 100 $\upmu \text{ m }$ and the actual location by the accumulated measured abrasion distances (error propagation). During the study, two different kinds of error propagation were observed: **a** in principle an excessive amount of abrasion (type 1, e.g., TB-4R), and **b** completely non-predictable progress of error propagation with partial compensation (type 2, e.g., TB-1R). A tendency towards insufficient amount of abrasion was not observed during the study

Apart from a few cross-sectional images, which were not correctly stored during photo-documentation, all resulting grinding surfaces were successfully dyed and digitally documented and were thus available for reconstructing a given specimen. For all serial cross-sectional images, their abrasion distance was documented, even in the few cases where image data was lost. The average abrasion distance of each sample is shown in Table 3. Additionally, the average error of abrasion, which was calculated as the difference between the achieved abrasion and the assumed equidistant abrasion of 100 $\upmu \text{ m }$, is listed for each sample. Furthermore, the accumulated error was calculated and its maximum value listed in Table 3. For this purpose, the actual (measured) location of each grinding image within the image stack was compared with the “theoretical” location. The theoretical or desired location of each image is defined by the assumed equidistant abrasion of 100 $\upmu \text{ m }$. Of course, the accumulated error is an abstract value since this error is in fact corrected by the integrated measurement step. Nevertheless, it underlines the importance and necessity of adequate measurement of the actual abrasion. As Fig. 7a, b show, there are two different kinds of error propagation. Since the error of abrasion is not evenly distributed, the reconstruction error varies strongly between several specimens and is therefore not predictable or correctable in any other way.

**Table 3:** Characteristic values and errors of serial cross-sectional preparation. The error of abrasion is determined as the difference between measured and adjusted abrasion. The maximum accumulated error is a theoretical value describing the worst dislocation of an image within the image stack of a specimen if equidistant abrasion had been used instead of the measured values. The reconstruction error describes the deviation of corresponding grooves within the fpVCT and the microgrinding images. The column type of error refers to the type of error propagation observed. See Fig. 9 for detailed information

Specimen (side)	Abrasion distance (mean $\pm$ SD) [$\upmu \text{ m }$]	Error of abrasion (mean $\pm$ SD) [$\upmu \text{ m }$]	Max. error (accumulated) [$\upmu \text{ m }$]	Reconstruction error top (apex) [mm]	Reconstruction error bottom [mm]	Type of error
TB-1L	101.6 $\pm$ 4.2	2.8 $\pm$ 3.4	277	0.1 $\vert$ 0.1 $\vert$0.2	0.0 $\vert$ 0.2 $\vert$ 0.3	1
TB-1R	100.0 $\pm$ 3.6	2.8 $\pm$ 2.3	-36	0.2 $\vert$ 0.2 $\vert$ 0.4	0.2 $\vert$ 0.3 $\vert$ 0.3	2
TB-3R	100.9 $\pm$ 6.6	4.4 $\pm$ 5.1	165	0.1 $\vert$ 0.1 $\vert$ 0.3	0.4 $\vert$ 0.3 $\vert$ 0.1	1
TB-4L	101.3 $\pm$ 4.8	3.3 $\pm$ 3.8	167	0.0 $\vert$ 0.1 $\vert$ 0.0	0.0 $\vert$ 0.1 $\vert$ 0.1	1
TB-4R	101.2 $\pm$ 3.5	2.5 $\pm$ 2.8	208	0.1 $\vert$ 0.1 $\vert$ 0.2	0.1 $\vert$ 0.1 $\vert$ 0.2	1
TB-5L	101.0 $\pm$ 5.7	3.9 $\pm$ 4.3	185	0.0 $\vert$ 0.2 $\vert$ 0.1	0.0 $\vert$ 0.0 $\vert$ 0.0	1
TB-5R	101.3 $\pm$ 5.0	3.5 $\pm$ 3.8	215	0.1 $\vert$ 0.2 $\vert$ 0.2	0.1 $\vert$ 0.0 $\vert$ 0.1	1
TB-6L	101.0 $\pm$ 5.1	3.6 $\pm$ 3.7	177	0.1 $\vert$ 0.3 $\vert$ 0.2	0.1 $\vert$ 0.1 $\vert$ 0.1	1
TB-6R	100.9 $\pm$ 4.7	3.4 $\pm$ 3.4	188	0.1 $\vert$ 0.1 $\vert$ 0.1	0.2 $\vert$ 0.0 $\vert$ 0.1	1

**Fig. 8:**
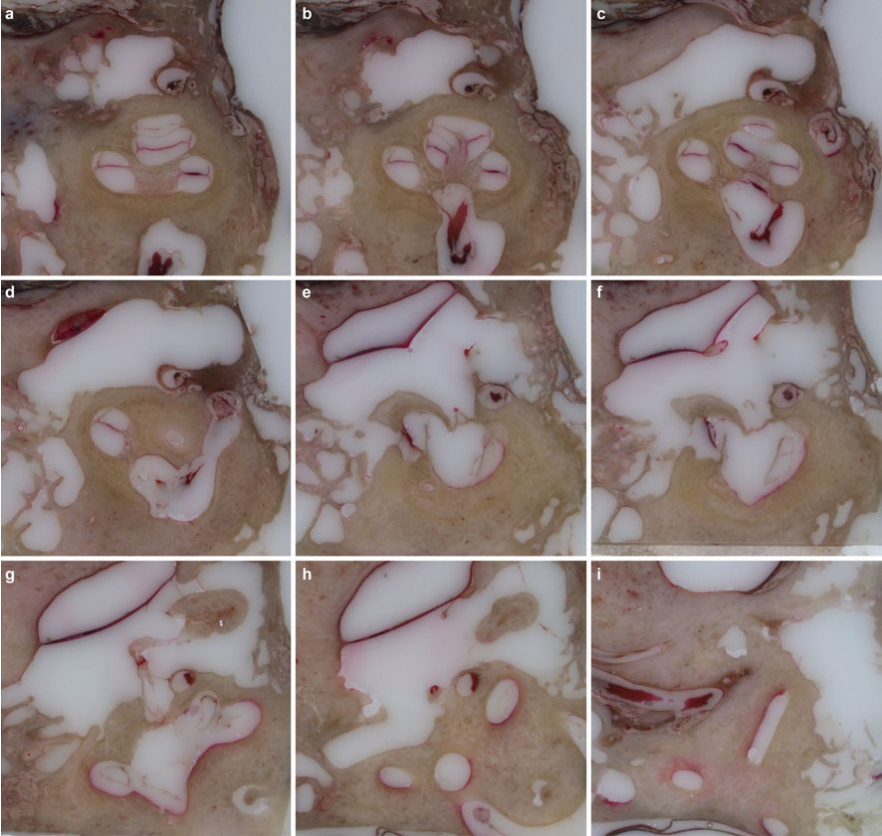
Successive series of cross-sectional images through the human inner and middle ear showing soft and bony tissue structures (TB-6L)

**Fig. 9:**
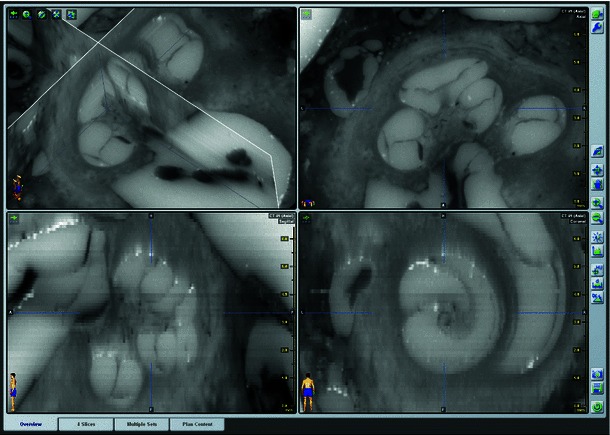
Three-dimensional Dicom data set of the histological microgrinding images. Screenshot of a Dicom data set loaded into the commercial clinical planning software iPlan ENT 2.6 (BrainLAB AG Feldkirchen, Germany) demonstrating the true three-dimensional character of the reconstructed histological cross-sectional images (3D histology). The subfigure on the *top right* shows the original histological image, whereas the subfigures in the *lower* panel correspond to reconstructed views (“sagittal” and “coronal” orientation). Even oblique reconstruction is possible (not shown)

**Fig. 10:**
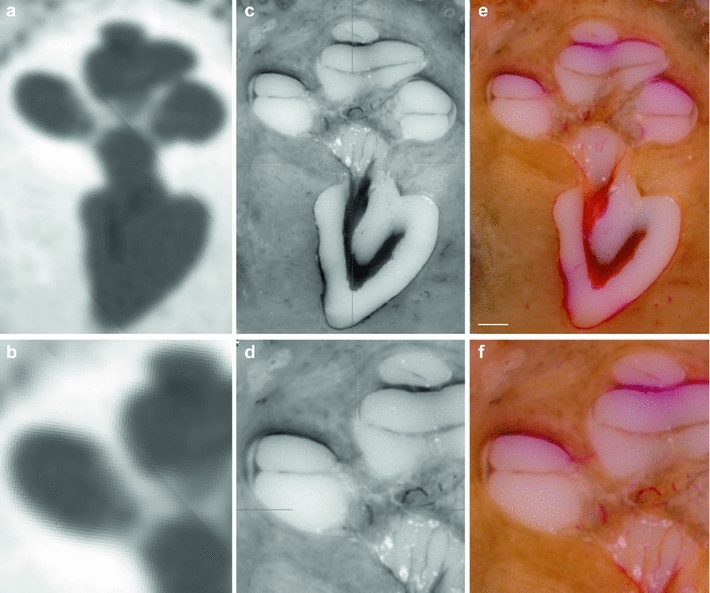
Corresponding views of the inner ear. **a**, **b** fpVCT, **c**, **d** 3D histological cross-sectional imaging after converting into the Dicom standard and **e**, **f** the original histological image. Radiology-based imaging provides only information about the bony boundary of the cochlea (**a**, **b**) and a blurred visualization in regions with thin bony structures containing a meshwork of vessels and neuronal tissue such as the modiolus with the cochlear nerve. In comparison, the presented procedure enables the visualization of both soft tissue structures such as the basilar membrane and stria vascularis, and thin bony structures like the osseous spiral lamina. Solely, neuronal structures cannot be completely preserved as the dehydration step, which causes their shrinking (**c**, **e**), is a necessary part of specimen preparation. The lower panel shows close-ups of the images (**a**), (**c**) and (**e**). (TB-1R, *scale bar* is 1 mm)

**Fig. 11:**
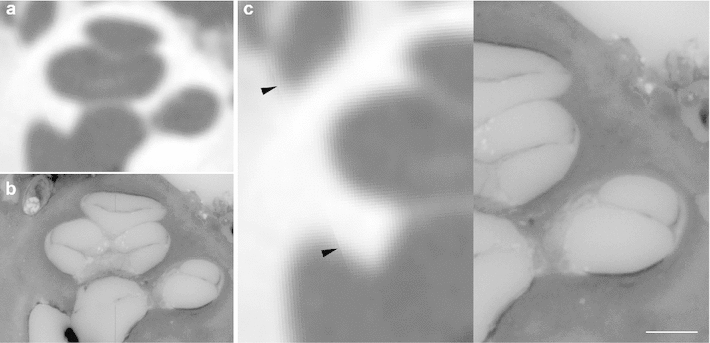
Image fusion of radiological and histological imaging. Image fusion of fpVCT data (**a**) and the 3D histology (**b**) of the same specimen for the comparative visualization of cochlear structures. While soft tissue structures such as the basilar membrane and stria vascularis—and also the osseous spiral lamina—are not visible in the radiological imaging method, these structures can be easily identified in the corresponding histological cross-sectional image. The artefact in the subimage (**c**, *black arrows*) is caused by the multiplanar reformation during image fusion which was performed in such a way that the histological image data remained unchanged, whereas the fpVCT data with isotropic voxels were transformed. (TB-1L, *scale bar* is 1 mm)

**Fig. 12:**
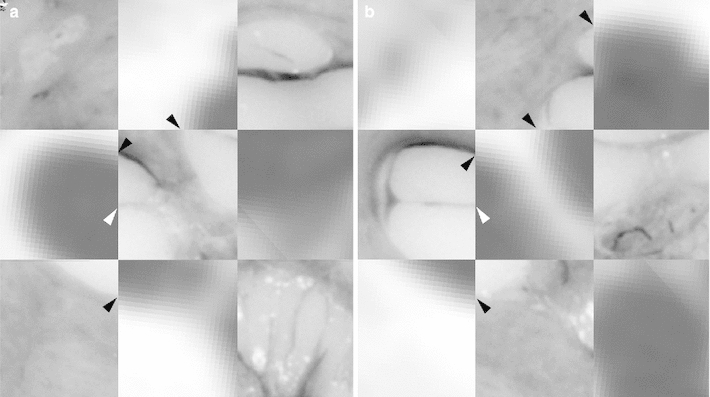
Image fusion to determine the reconstruction quality. Image fusion of a three-dimensional histological data set with the fpVCT data of the same specimen (see Fig. 10 for a total view of the microgrinding slice and the correlated layer in the fpVCT data). Due to the high degree of conformity between the shape of bony boundaries in both imaging methods (*black arrows*), it appears reasonable to assume that the locations of soft tissue structures such as the basilar membrane (attached to the lamina spiralis ossea, *white arrows*) can also be transferred with high accuracy. (TB-1R)

**Fig. 13:**
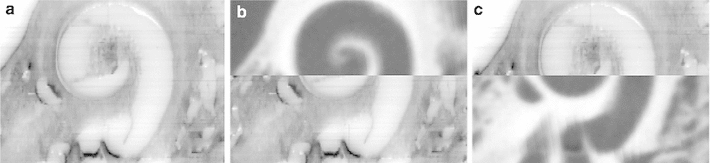
Orthogonal plane reconstruction due to true three-dimensional character of the micogrinding procedure. (**a**) Sagittal view of the same specimen as in Figs. 10 and 12. The abrasion distance is visible as slight bands. (**b**-**c**) Comparison with the corresponding fpVCT data after image fusion also shows the high accuracy. (TB-1R)

**Fig. 14:**
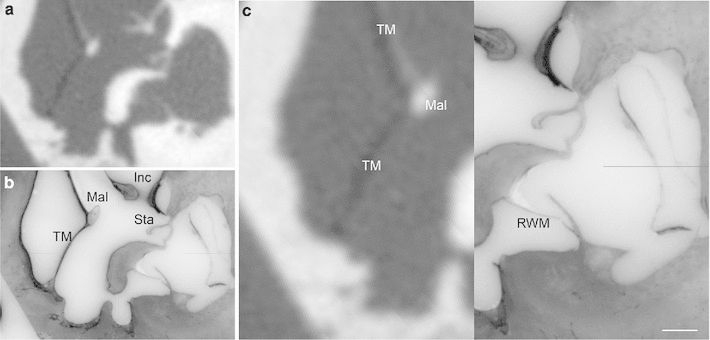
Histological imaging of the middle ear. Image fusion of fpVCT data (**a**) and the 3D histology (**b**) of the same specimen for the comparative visualization (**c**) of middle and inner ear structures around the round window. Conformity of bony boundaries in both imaging methods underlines the quality of the three-dimensional histological sample preparation procedure. *TM* tympanic membrane, *Mal* Malleus, *Inc*, Incus, *Sta* Stapes with footplate, *RWM* round window membrane. (TB-5L, *scale bar* is 1 mm)

After performing the grinding procedure, all serial cross-sectional images (Fig. 8) were successfully aligned to the corresponding base image via the described custom-made registration algorithm. The known distance between each of the three registration markers, provided by the data of the CNC drilling machine, allowed the determination of the particular pixel spacing value by dividing the metrical value by the determined distance in pixels. After cropping, each image stack could be successfully converted into the Dicom standard using the described Matlab algorithm. These data sets were loaded into iPlan ENT 2.6 (Fig. 9) and successfully merged with the already loaded fpVCT data (Figs. 10, 11, 12, 13, 14) using the intensity-based image registration algorithm of the software. Measuring the distances between the grooves in the serial—section images and in the fpVCT data revealed deviations for both imaging techniques that mostly lay between 0.0 mm and 0.1 mm (see Table 3) with some outliers of up to 0.4 mm.

In two cases, this verification step showed that the accuracy of the reconstruction procedure was not as high as expected and could thus be further improved. In the first case, the image fusion revealed the non-perpendicular position of the registration markers. While the grooves corresponded well within the base image, the grooves of the most distal part of the sample exhibited considerable deitns. The deviation of the registration markers from perpendicularity was determined and the resulting values used to correct image alignment during a second pass of the registration step. In the second case, the distance between two of the three registration markers varied from the other two. Firstly, therefore, a Dicom file set was generated using the mean value of all three distances to determine the pixel spacing and afterwards, a second one with only the two conformable values. After controlling both by means of the grooves, the more inaccurate was deleted.

Each of the samples underwent microgrinding as described, covering the complete middle and inner ear. Thus, as Table 1 shows, there are different numbers of images per individual sample. The number of images ranges between 132 and 185 per sample with an average value of 170 layers. In general, the number of slices depends on the sample size, its orientation within the epoxy resin and the chosen amount of abrasion and, directly influences the duration of preparation for a whole specimen.

After verification of the 3D reconstruction using the grooves, the image stack was cropped for a second time to reduce the amount of data for the following segmentation step. Only the relevant structures of the middle and inner ear were included since the grooves and registration markers were no longer needed. Depending on the size of the temporal bone specimen, the resulting cut varied between 994 $\times$ 880 and 1,030 $\times$ 1,102 pixels, as Table 1 shows. The wide range of image size is related to the chosen field of view for the cropping of the image stack. On the one hand, it depends on the size of each specimen showing the expected anatomical variation. On the other hand, the orientation of labyrinth in the epoxy resin cylinder significantly influences image size.

### Image fusion

Finally, the quality and accuracy of the presented sample preparation procedure is revealed by contrasting the serial cross-sectional images with the fpVCT data. Conformability of bony boundaries within the middle and inner ear shows that spatial positions and geometric dimensions of the specimens are preserved to a high degree (Figs. 11, 12, 13, 14). While the fpVCT imaging does not visualize intra-cochlear soft tissue structures, such as the basilar membrane and stria vascularis, these structures are clearly visible in the histological sections (Figs. 11, 12). Since the bony boundaries of, for instance, the cochlea and the auditory nerve correspond in both data sets, it is assumed that the soft tissue structures only visible in the microgrinding images also correspond in their location. Comparing histological images with the respective CT images additionally highlights the disadvantages of CT imaging, as for example, partial volume effects (Fig. 11). Comparable findings are provided by the visualization of middle ear structure, as can be seen in Fig. 14.

## Discussion

This paper presents a highly accurate procedure for imaging of the middle and inner ear based on a serial cross-sectional preparation. To our knowledge, this is the first time a detailed investigation into the accuracy of a three-dimensional reconstruction procedure of this nature has been included. Therefore, a well-known reference object with comparable dimensions to those of the middle and inner ear was used [78]. As could be shown, the method allows the dimensional stable 3D reconstruction from 2D histological sections. This facilitates the detailed visualization of small anatomical structures including the ossicular chain with its muscles, tendons and ligaments as well as the membranous intra-cochlear structures such as the stria vascularis and basilar membrane.

The development of an accurate histology-based imaging technique was necessary because standard medical imaging techniques such as MSCT or MRI do not yet offer the necessary high spatial resolution and tissue differentiation to visualize thin soft tissue structures. Therefore, a strategy called model-based imaging [89] is pursued in our further research on CAS in the field of cochlear implantation as well as simulation-based optimization of CI electrodes. This concept has potential for bridging a gap in customized medical imaging by adding missing information, provided by a more detailed database. This database is to be generated from the provided histological image stacks obtained by segmentation of all relevant bone and soft tissue structures in a representative number of human temporal bone specimens.

To overcome the above-mentioned drawbacks of clinically available imaging, several methods were investigated in experimental studies. In the context of high-resolution imaging, micro-CT scanners in particular have attracted increasing interest. In 1999, Vogel [106] presented a new approach for a three-dimensional modelling of the human inner ear using both X-ray and synchrotron radiation. Spatial resolutions as low as 30 and 11 $\upmu \text{ m }$ were achieved for the X-ray imaging and synchrotron radiation, respectively, which allowed adequate visualization of fluid and bony parts of the inner ear but provided no evidence of the basilar membrane or other intra-cochlear soft tissue structures. In 2004, Lane et al. [54] presented another study on imaging microscopy using a custom-made micro-CT scanner. By generating an isotropic data set of a cut temporal bone specimen with a spatial resolution of 20 $\upmu \text{ m }$, they were able to depict bone structures in detail. Thus, as already underlined in previous studies, micro-CT proves to be a suitable tool for high-density and high-contrast structures such as osseous spiral lamina [97] or the ossicular bones [16, 21, 57, 77, 84, 95]. High-resolution ossicular chain models, for example, are presented by Salih et al. [84] and freely available on the Internet.

For visualization of very small structures, however, the surrounding bone has to be minimized by specimen preparation because it absorbs a lot of radiation necessary for high-resolution imaging of interior structures [21]. Alternatively, the temporal bone specimen needs to be decalcified by the use of inorganic acid to decrease the difference in X-ray absorption between membranous and bony structures [75]. Thus, micro-CT scanning is not always non-destructive, as noted by Decraemer et al. [21]. Additionally, imaging of soft tissue is difficult [16, 92, 97, 106] and can only be realized by special approaches to enhancing the soft tissue contrast. Postnov et al. [75], for example, replaced the perilymph with air to increase the contrast of the basilar membrane. This baseline scan was digitally superimposed with a second scan after replacing the fluid in the cochlear scalae. Although even Reissner’s membrane became visible, as mentioned by the authors, the influence of this procedure on the dimensional stability of the affected membranous anatomy is as yet unknown. An apparently less problematic method seems to be the use of contrast agents such as the injection of osmium tetroxide ($\text{ OsO }_{4})$ [76, 105]. While osmium tetroxide is toxic and difficult to handle simpler and low-toxicity stains are described [68, 69] and successfully used for the human middle ear [2]. As for another drawback of micro-CT imaging—the noise of its raw data (especially in the context of subsequent segmentation)—the use of anisotropic diffusion methods for image denoising would appear to provide a suitable solution [76]. Finally, highly detailed anatomical models can be provided by combining micro-CT imaging with other modalities, as has been done by Buytaert et al. [16] for realistic middle ear modelling.

Focussing on soft tissue visualization, MRI is an object of research. Clinical application provides evidence that this method provides much better soft tissue differentiation than CT-based imaging, even though considerable restrictions in spatial resolution have to be accepted. Using high-field strength MR scanners and long acquisition time, the aim was spatial resolution in the range of micro-CT imaging [34, 49, 55, 85, 103]. Hence, it is often called magnetic resonance microscopy (MRM). These approaches are encouraging and help to promote a better understanding of complex human middle and inner ear anatomy.

Up to now, however, MRM has not provided the necessary high resolution for 3D modelling of the complex anatomy of the middle and inner ear [88]. Therefore, other non-destructive imaging methods have been developed, based on optical cross-sections of a transparent specimen. Although these techniques differ in detail, all use laser light which excites fluorescent light in the narrow region of a geometrically very thin laser sheet. A camera is focused on this thin laser sheet, processing only fluorescent light from the focal zone. In this way, 2D section images can be obtained [107]. By rotation or translation of the specimen, a well-aligned stack of images can be produced for further 3D modelling. All these related techniques are referred to as light-sheet-based fluorescence microscopy (LSFM) [15, 17, 86]. When virtual optical sectioning was first implemented, it was termed orthogonal-plane fluorescence optical sectioning (OPFOS) and used for imaging of guinea pig cochlea of [107, 108]. Subsequently this method has, for example, been applied to build-up a mouse cochlea database (MCD) [88] as well as a gerbil middle and inner ear model [16]. The MCD is available online and provides 2D serial image stacks as well as interactive 3D reconstructions of the ear.

As Table 4 show, complex procedures are necessary for specimen preparation, which include dehydration, decalcification, chemical steps to make the tissue transparent and the addition of fluorescent dye. Here, too, the resulting distortion and shrinking of the tissue influence the accuracy of the three-dimensional spatial reconstruction. An initial indication of the amount of shrinkage is provided by combining an OPFOS and a micro-CT model of the same ossicles from a gerbil specimen. A shrinking factor of 8.4 % was found [16]. Another challenge of OPFOS is the handling of neural tissue which cannot be rendered fully transparent and therefore must sometimes be removed [37]. However, for embryos and young mice (not older than two weeks), special clearing procedures are available that render complete brains (up to 2 cm in size) transparent [22].

**Table 4:** Experimental technologies for high-resolution imaging of the (human) middle and inner ear. Spatial resolution characterizes the size of each voxel. A cubed value indicates isotropic imaging; otherwise, the third value refers to differing slice thickness. If possible, physical resolution was quoted instead of pixel size of the image stacks after reconstruction. As few studies determine the spatial resolution using the modulation transfer function (e.g. 10 % MTF), comparability of these values is strongly restricted. An imaging technology is labelled as destructive if the geometry of the specimen becomes affected (according to [85]). In this context, a decalcification step was considered as destructive

Study	Imaging	Voxel size [$\upmu \text{ m }$]	Destructive	Sample preparation	Technical details, comments
	technique			* Specimen preparation	
				$\dagger$ Chemical fixation	
				$\ddagger$ Decalcification, dehydration	
				§ Staining, fluorescent dye, contrast agent	
				¶ Embedding, mechanical fixation	
*Micro-computed tomography* ($\mu CT$) *imaging*					
Vogel [106]	X-ray	$(60\,\upmu \text{ m })^{3}$ (30 $\upmu \text{ m })^{3}$	No	$\dagger$ Ethanol fixation	Custom-made $\upmu \text{ CT }$ scanner (150 kV, 200 mA)
	SR	(11 $\upmu \text{ m })^{3}$		$\dagger$ Ethanol fixation	SR source: HASYLAB at DESY, Hamburg, Germany (24 keV)
Spaendonck et al. [97]	X-ray	(8 $\upmu \text{ m })^{3}$	No	* Perfusion through round and oval window	Commercial $\upmu \text{ CT }$ scanner: Skyscan, Aartselaar, Belgium
				$\dagger$ 4 % Buffered paraformaldehyde followed by 0.01M phosphate- buffered saline (pH: 7.4)	
Decraemer et al. [21]	X-ray	(10 $\upmu \text{ m })^{3}$	Yes	$\dagger$ Frozen and air dried isolated cat stapes	Commercial $\upmu \text{ CT }$ scanner: Skyscan 1072, Aartselaar, Belgium (80 kV, 100 $\upmu \text{ A }$)
		(21 $\upmu \text{ m })^{3}$	No	$\dagger$ Human TB stored in water with a few drops of antiseptic	
		Up to (7.3 $\upmu \text{ m })^{3}$	Yes	* Isolated human ossicles, separately scanned	Image fusion using ICP algorithm
Lane et al. [54]	X-ray	(20 $\upmu \text{ m })^{3}$	No	No sample preparation	Custom-made $\upmu \text{ CT }$ scanner (35 kV, 50 mA)
Gao et al. [31]	SR DEI	(10.9 $\upmu \text{ m })^{3}$	Yes	$\dagger$ Perfusion of 0.9 % sodium and 4 % polyformaldehyde solution	Custom-made system for diffraction enhanced-based microtomography
				* Puncture of cochlea by clipping the footplate	Synchrotron source: 4W1A of BSRF, China (9.4 keV)
				$\dagger$ 30 % Saccharose formaldehyde solution	
				$\ddagger$ Decalcification	
				$\dagger$ 4 % Polyformaldehyde	
Postnov et al. [75]	X-ray	Up to ($9\,\upmu \text{ m })^{3}$	Yes	$\dagger$ 10 % Formalin solution (formol)	Commercial $\upmu \text{ CT }$ scanner: Skyscan 1076, Aartselaar, Belgium
				$\ddagger$ Decalcification in hydrochloric and nitric acid solution	
			Yes	* Suction of perilymph and introduction of air into the cochlea of a fresh temporal bone	Baseline scan with increased contrast
				* Replacing of the fluid using slight pressure from a vacuum pump	Second scan performed for digital superimposition
Uzun et al. [105]	X-ray	($10.5\,\upmu \text{ m })^{3}$	No	$\dagger$ Intra-cardiac perfusion of 3 % paraformaldehyde and 0.5 % glutaraldehyde	Commercial $\upmu \text{ CT }$ scanner: Skyscan 1072, Aartselaar, Belgium ($60\,\text{ kV },\,163\,\upmu \text{ A }$)
				§ 2 % $\text{ OsO }_{4}$ solution for three days	
Poznyakovskiy et al. [76]	X-ray	(10 $\upmu \text{ m })^{3}$	No	$\dagger$ Auditory bullae placed in HEPES–Hanks solution, 300 mOsm, pH: 7.34	Custom-made system using microfocal X-ray source (Feinfocus GmbH, Hamburg, Germany)
				$\dagger$ Perfusion of neutrally buffered 4 % Formaldehyde solution containing 3 mM $\text{ CaCl }_{2}$	
				§ 2 % $\text{ OsO }_{4}$ solution in 0.1 M sodium cacodylate buffer	
		$(4\,\upmu \text{ m })^{3}$		¶ Conservation in polyethylene glycol 400 same specimen	Scanning with reduced field of view and subsequent concatenation to a single data set
Sim and Puria [95]	X-ray	$(10.5\, \upmu \text{ m })^{3}$	No	* Harvested within two days and stored frozen	Commercial scanner: vivaCT 40, Scanco Medical AG, Switzerland ($55\,\text{ keV },\,145\,\upmu \text{ A }$)
Whiting et al. [115]	X-ray	(36 $\upmu \text{ m })^{3}$	No	$\dagger$ Formalin solution	Commercial scanner: $\upmu \text{ CT }$ 40, Scanco Medical AG, Bassersdorf, Switzerland ($70\,\text{ kVp },\,114\,\upmu \text{ A }$)
Lareida et al. [56]	SR	(4.3 $\upmu \text{ m })^{3}$(10 % MTF)	No	* Opening of round and oval window	SR source: HASYLAB at DESY, Hamburg, Germany (10.8 keV)
				$\dagger$ 4 % Paraformaldehyde with 0.1 % glutaraldehyde in 0.1M cacodylate buffer (pH: 7.4)	Binning to increase the contrast
				§ 1 % $\text{ OsO }_{4}$ in 0.05 M cacodylate buffer (pH: 7.4)	
				$\ddagger$ Dehydrated in a series of graded ethanol	
				¶ Embedded in Spurr’s low viscosity epoxy resin	
Shibata et al. [93]	X-ray	($12.2\,\upmu \text{ m })^{2} \times 77.5\,\upmu \text{ m }$	No	$\dagger$ 10 % Formalin solution, stored in 60 % ethanol	Commercial $\upmu \text{ CT }$ scanner: Actis+2, Corporation, Tokyo, Japan ($180\,\text{ kV },\,100\,\upmu \text{ A }$)
Glueckert et al. [33]	SR	$8.46\,\upmu \text{ m }$ (10 % MTF)	No	* Opening of oval window $\dagger$ 4 % Paraformaldehyde and 0.1 % glutaraldehyde in 0.1 M phosphate-buffered saline (pH: 7.4) § 1.5 % $\text{ OsO }_{4}$ for 60 min	SR source: HASYLAB at DESY, Hamburg Germany (35 keV) Binning to increase density resolution
Lee et al. 2010 [57]	X-ray	($19.5\,\upmu \text{ m })^{3}$	No	* Harvested within two days and stored frozen	Commercial scanner: vivaCT 40, Scanco Medical AG, Switzerland (45 keV, 145 $\,\upmu \text{ A }$)
Puria and Steele [77]	X-ray	($10.5\,\upmu \text{ m })^{3}$	No	–	Commercial scanner: vivaCT 40, Scanco Medical AG, Switzerland (55 keV, $145\,\upmu \text{ A }$)
Richard et al. [81]	X-ray	($16\,\upmu \text{ m })^{3}$	No	$\dagger$ 10 % Formalin solution	Commercial $\upmu \text{ CT }$ scanner: Scanco VivaCT-40, Scanco Medical, Zürich, Switzerland (55 keV)
		(16 $\upmu \text{ m })^{3}$			45 keV—for lower mineralized foetal specimen
Buytaert et al. [16]	X-ray	(8.5 $\upmu \text{ m })^{3}$	No	* Cardiac perfusion with physiological fluid	Custom-built $\upmu \text{ CT }$ scanner (120 kV, 58 $\upmu \text{ A }$)
Braun et al. [9]	X-ray	(5.9 $\upmu \text{ m })^{3}$	No	* Stored frozen	Commercial scanner: $\upmu \text{ CT }$ 40, Scanco Medical AG, Brüttisellen, Switzerland (70 kVp, $200\,\upmu \text{ A }$)
Rau et al. [80]	SR	(1.22 $\upmu \text{ m })^{3}$	Yes	$\dagger$ 10 % Paraformaldehyde in 0.1 M phosphate-buffered solution	SR source: 32-ID beamline, Advanced Photon Source, Argonne National Laboratory (25 keV)
				$\ddagger$ 10 % Ethylene glycol tetra-acetic acid in 0.1 M phosphate-buffered solution	
Salih et al. [84]	X-ray	(5.6 $\upmu \text{ m })^{3}$ to (34.4 $\upmu \text{ m })^{3}$	No	$\dagger$ 4 % formaldehyde	Custom-built $\upmu \text{ CT }$ scanner
Seifert et al. [92]	X-ray	41 $\upmu \text{ m }$ slices 81 $\upmu \text{ m }$ slices	No	–	Commercial scanner: XtremeCT, Scanco Medical AG, Zürich, Switzerland (36 different protocols)
*Magnetic resonance microscopy* (*MRM*) *and high-resolution magnetic resonance imaging* (*hrMRI*)					
Henson et al. [34]	MRM	(25 $\upmu \text{ m })^{3}$	No	$\dagger$ 4 % Phosphate-buffered formalin solution	7.1 T GE Omega System, RF coil
					58 h acquisition time
Salt et al. [85]	MRM	(25 $\upmu \text{ m })^{3}$	No	$\dagger$ Vascular perfusion of Heidenhain-Susa fixative with 37 % formaldehyde fixation, stored in 4 % phosphate-buffered formaldehyde	7.1 T GE Omega System, RF coil 13.5 h acquisition time
				§ Magnevist (commercial contrast agent)	
Koizuka et al. [49]	MRM	(35 $\upmu \text{ m })^{2}$	No	$\dagger$ In vivo fixation by cardiac perfusion of saline and 10 % acid formalin solution	7.05 T NMR spectrometer, RF coil 3D Imaging as scout view
		(25 $\upmu \text{ m })^{2} \times 250 \upmu \text{ m }$			cross-sectional visualization
Thorne et al. [103]	MRM	(25 $\upmu \text{ m })^{3}$ $(66\,\upmu \text{ m })^{3}$	No	$\dagger$ Differing fixation procedures using (vascular perfusion) Heidenhain-Susa fixative, 4 % phosphate-buffered formaldehyde and/or 3.1 % glutaraldehyde phosphate-buffered salt solution	7.1 T GE Omega System, RF coil 13.5 h acquisition time
				§$\,\text{ HgCl }_{2}$ solution to enhance contrast and Magnevist (contrast agent)	
Ghiz et al. [32]	hrMRI	(25 $\upmu \text{ m })^{3}$	No	$\dagger$ Vascular perfusion of Heidenhain-Susa fixative with 37 % formaldehyde fixation, stored in 4 % phosphate-buffered formaldehyde	7.1 T GE Omega System, RF coil 13.5 h acquisition time
				§${}\,\text{ HgCl }_{2}$ solution to enhance contrast and Magnevist (contrast agent)	
Silver et al. [94]	hrMRI	98 $\upmu \text{ m } \times 86\,\upmu \text{ m } \times 120\,\upmu \text{ m }$ 49 $\upmu \text{ m } \times 43\,\upmu \text{ m } \times 230 \,\upmu \text{ m }$ 23 $\upmu \text{ m }$	No	$\dagger$ 0.1 mmol/kg gadolinium solution	9.4 T Varian high-field MR scanner approx. 12 h acquisition time
					Resolution depends on the size of the used acquisition array size
					Imaging parameters not specified in the paper
Lane et al. [55]	MRM	(78.1 $\upmu \text{ m })^{3}$	No	$\dagger$ 4 % Phosphate-buffered formalin solution	3D fast spin-echo technique
				§ Phosphate-buffered (0.1 M) dilute gadolinium solution	9.4 T, 13 h acquisition time
*Orthogonal plane fluorescence optical sectioning microscopy (OPFOS)*					
Voie [108]$^{\mathrm{a}}$	OPFOS	Up to (16 $\upmu \text{ m })^{3}$	Yes	$\dagger$ 10 % Neutral-buffered formalin solution	Green 1.5 mW Ne–Ne laser
				$\ddagger$ Decalcified in 10 % ethylenediamine tetra-acetic acid (EDTA) solution and subsequent dehydration in ascending concentrations of ethanol	
				§ Spalteholz$^{\mathrm{b}}$ clearing technique to achieve tissue transparency and treatment with rhodamine-b isothiocyanate as fluorescent dye	
Hofman et al. [37]	OPFOS	10 $\upmu \text{ m }$ slices	Yes	See Voie [108]	Green 2.0 mW He-Ne laser
Santi et al. [88]	OPFOS	(5.435 $\upmu \text{ m })^{2} \times 20\,\upmu \text{ m }$	Yes	$\dagger$ 4 % Paraformaldehyde for 24 h	Commissioned service of Spencer Technologies, Seattle, WA, USA
				$\ddagger$ Decalcified in 10 % ethylenediamine tetra-acetic acid (EDTA) solution; subsequent dehydration in ascending concentrations of ethanol	
				§ Methyl salicylate/benzyl benzoate for tissue transparency and stained en block with rhodamine isothiocyanate	
		$$(1.205\,\upmu \text{ m })^{2} \times 10\,\upmu \text{ m }$$			
Hofman et al. [38]	OPFOS	2 $\upmu \text{ m }$ slices	Yes	$\dagger$ 10 % Formalin solution, neutrally buffered	Green frequency doubled nd:YVO4 neodymium-laser, 532 nm, 52 mW
				$\ddagger$ Decalcified in 10 % ethylenediamine tetra-acetic acid (EDTA) solution, dehydrated using seven-step ethanol series	
				§ Spalteholz$^{\mathrm{b}}$ clearing technique to achieve tissue transparency and treatment with rhodamine-b isothiocyanate as fluorescent dye	
Hofman et al. [39]	OPFOS	20 $\upmu \text{ m }$ slices	Yes	See Voie [108]	Green 100 mW Ventus type pulsed diode laser
Buytaert et al. [16]	OPFOS	–	Yes	* Cardiac perfusion with physiological fluid	Custom-built set-up using bidirectional light-sheet illumination [14, 15]
				$\dagger$ 10 % Neutral-buffered formalin bath	
				$\ddagger$ Decalcified in 10 % ethylenediamine tetra-acetic acid (EDTA) solution; dehydration using a slowly graded ethanol series	
				§ Slowly graded Spaltholz fluid$^{\mathrm{b}}$; stained with rhodamine-b.	

*DEI* diffraction enhanced imaging, *FOV* field of view, *ICP*iterative closest point (algorithm), *n.s.*, not specified, *RF*, radio-frequency, *SR* synchrotron radiation, *TB*, temporal bone

$^{\mathrm{a}}$ only 2D imaging, no 3D reconstruction performed

$^{\mathrm{b}}$ Spalteholz fluid: 5:3 solution of methyl salicylate and benzyl benzoate

The main advantage of the above-mentioned methods over histological serial sectioning is the non-destructive generation of serial cross-sectional images. Therefore, errors caused by loss of or damage to the slices, uneven slice thickness or inaccurate alignment can be excluded [97]. However, the low availability of the required technology is problematic. While micro-CT scanners are becoming increasingly commercially available, custom-made experimental set-ups are necessary for MRM and OPFOS. Furthermore, very specific know-how is needed on the part of the research groups, which normally has to be acquired over several years.

In comparison, a systematic and easy-to-follow procedure is presented which does not require expensive equipment. Additionally, it overcomes the mentioned drawbacks by providing visualization for both bony and soft tissue. Moreover, histological section preparation is still considered the gold standard for the evaluation of new imaging techniques as performed, for example, to evaluate the OPFOS [39] and MRM [71], or to compare the quality of MSCT imaging [117] and micro-CT imaging [75, 97], with histological sections.

Especially in the field of cochlear implant electrode evaluation using temporal bone specimens, serial cross-sectional imaging is still an essential tool with regard to the preservation of intra-cochlear soft tissue structures [1, 12, 48, 59, 82, 100, 113] as well as for the development of new imaging techniques for quality control after cochlear implantation [3, 4, 8, 43, 50]. For this purpose, the presented procedure opens up new possibilities, since it enables spatially accurate image fusion of post-experimental imaging and histological section preparation. To date, however, only an approximate comparison has been possible because corresponding views have to be chosen and aligned manually.

The most serious disadvantages pointed out by the published studies on histological serial cross-sectional imaging are artefacts caused by tissue preparation and the slicing technique, as well as errors due to inaccurate image alignment or non-equidistant slice thickness [34, 54, 97]. In fact, serial-section preparation can generally be seen as a procedure including some or all of the following steps, each of which is prone to errors: specimen preparation (e.g. dehydration, decalcification, fixation, trimming, embedding), sectioning (e.g. slicing, milling or grinding), digitalization (e.g. scanning, photographing) and several steps involved in image processing (e.g. image registration, filtering, segmentation, 3D reconstruction). Particularly noteworthy in this context is the fact that none of the studies about serial cross-sectional preparation known to us investigated the accuracy of the reconstruction procedure. This study is, therefore, focused both on the development of a highly accurate preparation and reconstruction procedure *and* its quantification.

This includes reducing the number of chemical steps in specimen preparation, especially the omission of decalcification. The preservation of bony structures as a rigid skeletal frame in combination with rigid embedding in epoxy resin also improves the spatially accurate reconstruction of soft tissue structures. Most of them are in close proximity and connected to bony structures which define and fix their spatial arrangement, such as the basilar membrane which stretches from the osseous spiral lamina to the outer bony wall via the radial fibres of the spiral ligament. This is an important advantage of the chosen procedure compared with non-rigid embedding methods such as embedding in paraffin. Although sectioning by microtome after paraffin embedding is also available in our laboratory, the introduced procedure was preferred because of the above-mentioned advantages. In our experience, it is not possible to perform an accurate 3D reconstruction of paraffin slices due to their inevitable wrinkling. Besides the advantage of a reduced risk of tissue deformation, other hard tissues (e.g. dental enamel) and metallic components of, for example, implants can thus be included in sample preparation that applies embedding in epoxy resin and microgrinding. Therefore, this procedure is of special interest for the evaluation of cochlear implant electrode insertion with regard to the determination of soft tissue preservation and the spatial relation between tissue and implant.

For image registration, external markers were chosen since perpendicular drilling using a CNC device provides the highest possible accuracy. An intensity-based registration algorithm was tested which, however, revealed an increasing registration error throughout the image stack, observable via the visible—though unused—registration markers. One reason for this might be the use of each image’s preceding image for alignment instead of a common base image. Hence, for further improvement of image stack alignment, a custom-made algorithm was developed which automatically detects the location of the registration markers via a two-stage process [78]. This enabled errors caused by manual image alignment to be excluded.

The most crucial aspect in performing serial cross-sectional imaging is to ensure equal image distances. A special specimen holder was therefore developed which allows precise definition of the desired abrasion via a fine thread and an abrasion-resistant hard ceramic ring. As a precaution, each abrasion was additionally measured. The analysis of these data shows that, in spite of the mechanical limitation of abrasion, a non-negligible error occurs. The measurement results provided an opportunity to compensate for this error during the construction of the Dicom headers. This was facilitated by the flexible Dicom standard which does not require equal image distance. Instead, the specific location within the stack can be assigned to each image. Possible reasons for the variation of abrasion distance might be the clearance of the thread and the manual pressing of the specimen holder on the grinding disc. It thus seems possible to remove material in the micrometre range even below the level of the hard ceramic ring.

Therefore, the determination of the de facto abrasion would appear to be necessary (as long as a more accurate adjustment and the achievement of equidistant abrasion are not available) for several reasons. Firstly, the error of abrasion is not uniformly distributed so that it is not compensated for stochastically. Secondly, proper measurement of the reconstructed total length does not allow the conclusion that all cross-sectional images within the stack are correctly located. As Fig. 7b shows, the error of abrasion remains undetectable without measuring. Thirdly, continuous measurement can improve the achieved accuracy since the presented results are based on the correction of insufficient abrasion by means of an additional grinding step. Fourthly, the deviation from equidistant abrasion will become more crucial if the abrasion is further reduced to enable more regular voxels.

The results achieved with the reference object confirm the practicability and dependability of the developed procedure. Deviations of less then $0.4^{\circ } \,\pm \, 0.3^{\circ }$, taking into account shearing and tilting, show that only negligible distortion occurs during reconstruction. Likewise, an error in the range of 2.0 % for the reconstruction of lengths seems to be acceptable and tolerable for the underlying intention. Especially with regard to the aim of constructing an average anatomical atlas, these deviations are below inter-individual variations and thus compensated for by the averaging process. Comparison with outcomes of other 3D reconstruction procedures was not possible because, thus far, accuracy has not been reported in the literature. Although the results achieved with the reference object cannot be completely transferred to the embedding and reconstruction of tissue samples, the additionally performed image fusion confirms the consistency of the procedure even in this more complex context. Throughout all specimens, the discrepancy of the groove position within the fpVCT and the microgrinding image stack was not higher than 0.2 mm. Finally, the nearly equal shape of bony boundaries within the sample in both imaging methods can be considered admissible evidence for the accuracy of the introduced procedure. Remaining minor deviations are negligible because, finally, the approach of model-based imaging uses average values for several specimens, as mentioned above. Additionally, the intensity-based image fusion algorithm of iPlan is only performed on a limited area in the centre of the data set. Thus, the highest deviations occur at the image borders and are recorded by the measurement procedure. In the centre of the data, where the relevant anatomical structures are located, the error is very likely to be smaller. However, the use of fpVCT imaging as reference imaging modality is not an optimum solution as the resolution is limited. In general, it is recommended that the resolution of the reference standard is at least one dimension smaller than the investigated imaging procedure. This is not the case if comparing histological cross-sectional microgrinding preparation with fpVCT. For further investigations, the use of micro-CT imaging should be taken into consideration.

**Fig. 15:**
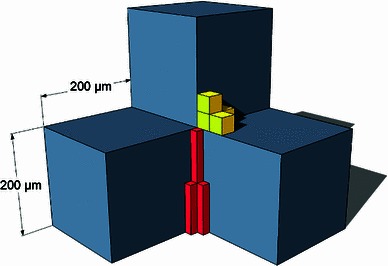
Comparison of the voxel size of different image modalities. The *grey blue* voxels represent the physical resolution of the used flat-panel volume computed tomography with an isotropic edge length of 200 $\upmu \text{ m }$. The *yellow cubes* symbolize the resolution of a micro-CT scanner used in former studies with a voxel size of approximately 30 $\upmu \text{ m }$. Finally, the red cuboidal volumes represent the voxel size of the presented microgrinding procedure with a length of 100 $\upmu \text{ m }$ in the direction of abrasion and approximately 16 $\upmu \text{ m }$ in the grinding plane

With an approximate pixel size of 16 $\upmu \text{ m }$ in each cross-sectional image, the achieved imaging quality is high enough for adequate and detailed visualization of the relevant anatomy. Voxel size in the x-y plane can be increased still further by improving the magnification of the microscope and the resolution of the digital camera. In contrast, the voxel size in the z-direction (100 $\upmu \text{ m }$) is currently proportionally much lower, although it is twice as good as in fpVCT (approx. 200 $\upmu \text{ m }$ isotropic voxel size, see Fig. 15) or highly improved compared with clinical MSCT (approx. 600 $\upmu \text{ m }$ in the z-direction). As a consequence of the microgrinding procedure, voxels are not isotropic. This is a disadvantage for multiplanar reconstruction (MPR), as image quality will be altered. This applies especially to the basilar membrane, which is the most relevant anatomical structure but whose size is of the same order of magnitude. In principle, the differing edge lengths of a voxel can be equalized by decreasing abrasion distance, even though isotropic values are barely achievable. Unfortunately, the microgrinding procedure is very time-consuming. In addition to the already considerable amount of time needed for the preparation of one specimen with 100 $\upmu \text{ m }$ abrasion, the necessary increase of resolution in the z-direction is another strong argument for automating the procedure. This particularly relates to the mechanical steps, including adjustment of abrasion, microgrinding, measurement and digital documentation. Taking these facts into account, the procedure requires an automated system comparable to the one used in [83].

Despite the quantitative determination of reconstruction accuracy and the carefully selected preparatory steps, it cannot be ruled out that the procedure influences the geometric dimensions of tissues. As the focus of this study was on the accuracy of the 3D reconstruction of sequential cross-sectional histological imaging, the chosen procedure does not allow the determination of the shrinkage and distortion caused by the specimen preparation steps, because fpVCT images were acquired after embedding. Therefore, this study does not provide information about the extend to which histological sectioning data deviate from the unprepared fresh specimens. This needs to be addressed in a future study utilizing additional imaging before commencing the specimen preparation and embedding procedure. For example, the shrinking of neuronal structures, this such as the vestibulocochlear nerve is conspicuous, this probably being caused by the dehydration. However, this is a common problem in all imaging techniques that require sample preparation. As shown in Table 4, most of the published procedures require one or more of the critical chemical sample preparation steps such as dehydration or decalcification. Likewise, if performing micro-CT imaging using contrast agents or OPFOS and rendering the tissue transparent (mostly via invasive perfusion through the round or oval window), integrity and dimensional preservation of the tissue cannot be ensured completely. Due to processes like these, none of the mentioned high-resolution imaging techniques can be applied to living humans. This underlines once more the importance of model-based imaging as a powerful tool to compensate missing anatomical details in the clinically available modalities (Table 5).

**Table 5:** Serial cross-sectional preparation techniques for high-resolution imaging of the (human) middle and inner ear. 3D indicates three-dimensional reconstruction of the cross-sectional images performed in the study; otherwise, only cross-sectional images were acquired (2D)

Study	Preparation technique	Spatial resolution $\times$ Slice thickness [$\upmu \text{ m }$]	2D/3D	Sample preparation	Technical details, comments
				* Specimen preparation $\dagger$ Chemical fixation	* Sectioning/milling technology
				$\ddagger$ Decalcification, dehydration	$\dagger$ Image registration method
				§ Staining, fluorescent dye, contrast agent	
				¶ Embedding, mechanical fixation	
Hofman et al. [39]	HPMA		3D	$\dagger$ 10 % Neutral-buffered formalin solution	* Microm microtome, Heidelberg, Germany
				$\ddagger$ Decalcified in 10 % ethylenediamine tetra-acetic acid (EDTA) solution and subsequent dehydration in ascending concentrations of ethanol	$\dagger$ Perpendicular drill holes for image registration, manual image alignment
				¶ Embedding in ethanol:2-hydroxypropylmethacrylate (HPMA) and dimethylphenylamine as catalyst	
				§ Sections stained with toluidine blue and contrast stained with basic fuchsine	
Li et al. [62]	Celloidin	(8.059 $\upmu \text{ m })^{2} \times 20 \upmu \text{ m }$	3D	$\dagger$ Formalin solution $\ddagger$ Ethylenediamine tetra-acetate (EDTA) decalcification and dehydration ¶ Celloidin solution hardened in cedar oil	* Sliding microtome (Leica Jung Histoslide 2000, Leica Microsystems CMS GmbH, Wetzlar, Germany)
					$\dagger$ Four perpendicular reference holes, automated image alignment
Li et al. [61]	Celloidin	(12.66 $\upmu \text{ m })^{2}$	3D	$\dagger$ Formalin solution	$\dagger$ Pair by pair manual image alignment
		$\quad \times$ 20 $\upmu \text{ m }$		$\ddagger$ Ethylenediamine tetra-acetate (EDTA) decalcification	
				¶ Celloidin solution	
Liu et al. [63]	Cryosection	30 $\upmu \text{ m }$	3D	$\dagger$ 4 % Paraformaldehyde solution and 0.9 % salt solution * Puncture of round and oval window	* Serial sections parallel to modiolus $\dagger$ Perpendicular holes for image alignment, automated image alignment, manual fine adjustment
				$\dagger$ Immersion of 30 % sucrose-formaldehyde solution	
				$\ddagger$ Decalcification with Plank’s solution	
				§ Stained by 1.5 % silver nitrate solution and subsequent deoxidation by pyrogallol	
				$\ddagger$ Dehydration	
				¶ Infusion of blue gelatine solution ($60^{\circ }\text{ C }$) and finally quick freezing in liquid nitrogen	
Sørensen et al. [96]$^{\mathrm{a}}$	Cryosection	(50 $\upmu \text{ m })^{3}$	3D	$\dagger$ Immersion in cold 10 % carboxymethylcellulose and removal of air by vacuum, deep freezing using carbon dioxide ice	* 450 MP cryomicrotome (LKB, Sweden), 25 $\upmu \text{ m }$ section thickness, digital photography of every second section
					$\dagger$ Vertical drill holes as reference points, custom-made image registration algorithm
Spaendonck et al. [97]	Paraffin	$10\,\upmu \text{ m }$	2D	* Perfusion through round and oval window $\dagger$ 4 % buffered paraformaldehyde followed by 0.01M phosphate-buffered saline (pH: 7.4)	* Serial sections parallel to modiolus
				$\ddagger$ Decalcified in 5 % ethylenediamine tetra-acetic acid (EDTA) solution, dehydrated in a graded alcohol series	
				¶ Paraffin embedding	
				§ Sections stained with hematoxylin-eosin	
Sun et al. [101]$^{\mathrm{b}}$	Celloidin	$200\,\upmu \text{ m }$	3D	$\dagger$ Aldehyde solution $\ddagger$ 10 % Nitric acid for decalcification and dehydrated in a graded alcohol series ¶ Penetration of celloidin solution (3 %, 6 %, 12 %) using a vacuum bottle § Sections stained with hematoxylin-eosin or Masson’s trichrome	* Sliding microtome (AO 860, American Optical Corporation), 20 $\upmu \text{ m }$ section thickness, every tenth mounted on glass slides, stained and scanned with 1,200 ppi
					$\dagger$ Four perpendicular reference holes drilled, filled with permanent ink, manual image alignment
Wang et al. [112]	Celloidin	100 $\upmu \text{ m }$	3D	$\dagger$ Formalin solution $\ddagger$ Decalcified using 5 % trichloroacetic acid ¶ Celloidin solution § Sections stained hematoxylin-eosin	* Serially sectioned, 20 $\upmu \text{ m }$ slice thickness, every fifth section stained , mounted on glass slides and scanned
					$\dagger$ Pair by pair interactive image alignment

$^{\mathrm{a}}$ Data used by [89, 111]

$^{\mathrm{b}}$ Same method used by [27–29, 102]

HPMA—hydroxypropylmethacrylate

The presented imaging method based on a highly accurate serial cross-sectional preparation technique provides the groundwork for a new generation of middle and inner ear models. These include all necessary bone and soft tissue structures, such as are necessary for FEA. Furthermore, in the context of cochlear implant treatment, this procedure creates new possibilities for the evaluation of intra-cochlear electrode placement. By means of such an accurate correlation of CT-based and histological imaging during temporal bone studies, the quality management of cochlear implant surgery using clinical imaging can be further improved and established. Finally, complex models for improved implant development based on computer-aided design (CAD) and computer-aided optimization (CAO) can be generated from the provided image series using common segmentation techniques.

However, our prior motivation is the use of these data for segmentation and, finally, for the development of an anatomical database (atlas) of the human middle and inner ear. For this purpose, additional specimens shall be prepared according to the described procedure to calculate a statistically confirmed anatomy atlas employing average values from a representative number of data sets. This is the basis for the designated implementation of model-based imaging to achieve adequate preoperative planning of computer- and robot-assisted approaches within cochlear implant surgery. Only if the problem of imaging for preoperative planning is solved satisfactorily can highly accurate assistance devices be established for the surgical procedure, as mentioned in the Introduction. In particular, the accurate opening of the cochlea (cochleostomy) and the insertion of the electrode array into the scala tympani require adequate determination of the location of the basilar membrane during planning and performing of the minimally invasive procedure. Of course, the implantation of a cochlear device is only one example among a wide range of surgical approaches which can benefit from the three-dimensional, highly accurate histological imaging method, presented here.

## Conclusion

There are multiple modalities in temporal bone imaging which are widely present in clinical routine and used for diagnostics in anatomical malformation, tumours or inflammatory diseases as well as for preoperative planning for interventions at the lateral skull base. However, imaging—of both soft and bony tissues—with high spatial resolution for highly accurate surgical procedures such as cochlear implantation remains a challenge. As part of our research in the field of cochlear implant surgery using mechatronic assistance, we pursue a model-based approach for preoperative planning to fill this gap in clinically available patient-specific imaging. Therefore, a database (atlas) of the human middle and inner ear should be generated using high-resolution images that include all necessary bony and soft tissue structures, especially the location of the basilar membrane.

For this reason, we developed and presented a highly accurate serial cross-sectional preparation technique. Accuracy was determined for the first time using a well-known reference object which has successfully proven its usability. The systematic and easy-to-follow preparation technique provided allows image fusion of the reconstructed image stack with conventional (imaging) modalities. The application of the procedure to human temporal bone specimens and its image fusion with fpVCT data underlined the spatial accuracy achieved.

The presented procedure opens up new possibilities in the fields of model-based preoperative planning, image-guided and computer-aided surgery, and biomechanical FEA by providing highly detailed and accurate imaging of both bony and soft tissue structures.

## Footnotes

1 Also known as intra-operative navigation systems.

2 Anonymized temporal bones were used for this study; thus, no approval by the appropriated ethics committee at Hannover Medical School was necessary. As a consequence, no patient-specific data are available, such as sex, age or pre-existing conditions.
